# The impact of land use change on mycorrhizal fungi and their associations with rodents: insights from a temperate forest in Mexico

**DOI:** 10.1007/s00572-025-01210-x

**Published:** 2025-05-08

**Authors:** Margarita Gil-Fernández, Alexandra J. R. Carthey, Eduardo Mendoza, Oscar Godínez-Gómez, M. Cristina MacSwiney G., Arnulfo Blanco-García, Christian A. Delfín-Alfonso, Johannes J. Le Roux

**Affiliations:** 1 School of Natural Sciences, Macquarie University, New South Wales, 2109 Australia; 2 Posgrado en Biología Integrativa, Instituto de Investigaciones Biológicas, Universidad Veracruzana. Luis Castelazo Ayala Avenue, Industrial Ánimas, Xalapa, 91190 Veracruz, Mexico; 3 Laboratorio de Vertebrados, Instituto de Investigaciones Biológicas, Universidad Veracruzana. Luis Castelazo Ayala Avenue, Industrial Ánimas, Xalapa, 91190 Veracruz, Mexico; 4 Instituto de Investigaciones sobre los Recursos Naturales, Universidad Michoacana de San Nicolás de Hidalgo, San Juanito Itzicuaro Avenue, Nueva Esperanza, Morelia, Michoacán 58330 México; 5 Department of Wildlife Ecology and Conservation, School of Natural Resources and Environment, University of Florida, Gainesville, FL 32618 USA; 6 Centro de Investigaciones Tropicales, Universidad Veracruzana, José María Morelos y Pavon 44, Centro, Xalapa, Veracruz, 91000 México; 7 Facultad de Biología, Universidad Michoacana de San Nicolás de Hidalgo. Francisco J. Múgica Avenue, Ciudad Universitaria, 58060 Morelia, Michoacan Mexico

**Keywords:** Arbuscular mycorrhizal fungi, Ectomycorrhizal fungi, DNA barcoding, Fungal seasonality, Mycorrhizal fungi vectors

## Abstract

Ecosystem functioning is influenced by biological diversity, ecological interactions, and abiotic conditions. Human interactions with ecosystems can cause major changes in how they function when involving changes in the vegetation cover and structure (i.e., land use change). This study examines how land use change affects the diversity of arbuscular mycorrhizal fungi (AMF) and ectomycorrhizal fungi (EMF) in soil and rodent scats in temperate forest sites. We collected soil and rodent scat samples at five paired sites (i.e., disturbed vs. undisturbed) in Michoacan, Mexico. We identified 112 putative mycorrhizal fungi species using DNA barcoding based on partial internal transcribed region 1 (ITS) sequences. We found a higher richness of EMF in undisturbed soil samples compared to disturbed soil samples and a higher AMF diversity in rodent scat samples from disturbed than undisturbed sites. Scat samples had a high incidence of both AMF (75%) and EMF (100%). We found significant differences in the diversity of both AMF and EMF depending on the rodent species associated with them. We also found a higher diversity of EMF in scats in the wet season than in the dry season. We also report, for the first time, associations between *Sigmodon hispidus* and numerous AMF and EMF species. Overall, our study highlights the role of rodents as important dispersal vectors of mycorrhizal fungi, particularly for EMF that could be essential to build up mycorrhizal fungi spore banks in disturbed forests.

**Supplementary Information:**

The online version contains supplementary material available at 10.1007/s00572-025-01210-x.

## Introduction

Temperate forests provide various ecosystem services, including carbon sequestration, water provisioning, climate regulation, and biodiversity maintenance (Martínez Pastur et al. 2018). However, forests are also exposed to more frequent and intense anthropogenic disturbances (e.g. pollution and deforestation), that pose a significant threat to their functioning (Battisti et al. 2016; Newman 2019). While forests have evolved alongside natural disturbance regimes such as droughts, windstorms, and fires (Johnstone et al. 2016), they now increasingly face the combined effects of natural and modern anthropogenic disturbances (Lucash et al. 2018; Newman 2019). As a strategy to partly increase the level of tolerance to environmental stress associated with disturbance, most plants associate with symbiotic mycorrhizal fungi (Ferrol and Lanfranco 2020; Amalia et al. 2021). These associations are essential for nutrient cycling and enhancing the uptake of water and nutrients by plants, as well as protecting them against pathogens (Zengpu et al. 1994; Mukerji et al. 2012; Sarkar and Sadhukhan 2023). Land use change is one of the most impactful anthropogenic disturbances, defined as changes in natural vegetation cover to accommodate different human uses (Potapov et al. 2022). These changes in vegetation often involve a complete turnover of the plant communities (Lammel et al. 2021). Land use change is globally prevalent, impacting nearly 75% of the Earth’s surface (Winkler et al. 2021). The interactions between fungi and plants may be crucial in mediating the responses of plants to anthropogenic land use change (Kałucka and Jagodziński 2016; Tedersoo et al. 2020), yet our understanding of these responses remains rudimentary.

Globally, arbuscular mycorrhizal fungi (AMF) and ectomycorrhizal fungi (EMF) are the most studied types of mycorrhizal fungi. In disturbed areas, the community composition of mycorrhizal fungi is closely related to, and defines, the vegetation structure (Tedersoo et al. 2020). In response to land use change, AMF communities tend to have a low richness (House and Bever 2018) or become dominated by species that are associated with herbaceous plants that dominate when woody EMF-associated plants have been removed (Clavel et al. 2024). Comparatively, the richness of EMF communities tends to remain the same or decrease, depending on the type of disturbance that an area has experienced (Kranabetter et al. 2017). Generally, land use change leads to the turnover of mycorrhizal fungi communities, with specialist mycorrhizal fungi disappearing because of the loss of their host plants (van der Heyde et al. 2017; Hewitt et al. 2022). However, these changes are not always detected in DNA barcoding studies, due to the presence of relict mycorrhizal fungi DNA in soils (Carini et al. 2016). This highlights the need for including diverse sample types (e.g., soil, litter and scats) when studying the responses of mycorrhizal fungal communities to disturbance (Bradshaw et al. 2022).

Local environmental conditions, including humidity, precipitation, and soil characteristics, such as pH, largely drive the composition of mycorrhizal fungal communities (House and Bever 2018). Therefore, land use changes affecting these conditions could, in turn, impact mycorrhizal fungal communities and their plant hosts (Lewandowski et al. 2015). Human-induced disturbances to soil structure also affect mycorrhizal associations through the destruction of hyphal networks (Chagnon et al. 2013). Likewise, the loss of vegetation due to clearing leads to the heating and drying of soils, adversely affecting the diversity and structure of mycorrhizal fungi (Tedersoo et al. 2014). Furthermore, the use of chemical products in soils, such as pesticides and fungicides, can have a negative effect on mycorrhizal fungi diversity or inhibit the formation of plant mycorrhizal associations (Shukla et al. 1996; Trappe and Strand 1969; Trappe 1984; Chakravarty and Sidhu 1987; Laatikainen and Heinonen-Tanski 2002; Jin et al. 2013).

The diversity and structure of mycorrhizal fungal communities are also influenced by fungal spore dispersal, regardless of habitat disturbance levels (Peay et al. 2012; Dundas et al. 2018; Paz et al. 2021; Elliott et al. 2022b). Both AMF and EMF require animals, and to a lesser extent, wind and water, to disperse their spores (Borgmann-Winter et al., 2023; Aguirre et al. 2021; Bueno &Moora 2019; Kivlin et al., 2011). Among mammals, rodents stand out as one of the most diverse groups of dispersers of mycorrhizal fungi (Elliott et al. 2022b). Rodents disperse mycorrhizal fungi through direct ingestion of the fruiting bodies of EMF (i.e., mushrooms and truffle-like fungi) and the sporangia of AMF, or through incidental ingestion when feeding on plant material (Bueno and Moora 2019; Verde Arregoitia and D’Elía 2021). Moreover, both types of fungi can be dispersed externally by attaching to animals (Vašutová et al. 2019). Due to their habits, many rodents have the potential to disperse spores to favourable sites for their germination, such as areas with shrubby vegetation or inside their protective burrows (Warner et al. 1987). This function can be especially important in disturbed areas to facilitate plant succession (Aguirre et al. 2021). For example, rodent scats loaded with mycorrhizal fungal spores have been found in post-logging regeneration sites, which could help with temperate forest regeneration (Schickmann et al. 2012).

Some rodent species consume and disperse more mycorrhizal fungi than others. For example, *Myodes glareolus* in central Europe, disperse more spores and a more diverse set of mycorrhizal fungi species than other sympatric rodent species (Schickmann et al. 2012). The same rodent species has also been found to prefer truffle-like *Octaviania* and *Hysterangium* species over other types of fungi (Komur et al. 2021). Even generalist rodents, like *Peromyscus maniculatus*, play a key role in dispersing rare mycorrhizal fungi species (Stephens and Rowe 2020). Therefore, rodent activity has the potential to affect mycorrhizal fungi diversity and, in turn, the structure and function of forests (Stephens and Rowe 2020).

Rodents feed on fungi throughout the year, however, in the case of EMF, the production of fruiting bodies is strongly linked to rainfall and temperature (Sugiyama et al. 2020; Heklau et al. 2023). For example, the fruiting bodies of epigeous EMF (e.g., mushrooms) are more abundant in wetter and warmer seasons than in dryer and cooler seasons (Boa 2004; Pilz et al. 2003; Quiñónez-Martínez et al. 2014). Similarly, the production of fruiting bodies of hypogeous EMF (e.g., truffle-like structures) has peaks coinciding with wet seasons (e.g., Gómez-Reyes et al. 2018). As fruiting bodies are especially attractive as food for rodents, the seasonal peak of their production would typically coincide with a peak in their consumption (Elliott et al. 2022a), even when the consumption of fruiting bodies may occur all year round (Vernes et al. 2015; Nest et al. 2023). Yet, the diversity of consumed fungi could be determined by geographic variation in environmental variables, which leads to differences in fungal phenology (Elliott et al. 2020). Similar to EMF, the diversity of AMF is closely linked to rainfall and soil moisture (Ji et al. 2021; Dickey and Fordyce 2024; Veras et al. 2024), affecting their availability as food resources to rodents, whether via direct feeding on AMF sporangia or incidental ingestion. Given the high seasonal variability of interactions between mycorrhizal fungi and rodents, it is important to establish baseline data for specific areas to detect shifts in these in response to climatic stress conditions (Fernandez et al. 2023; Nieves et al. 2024).

Disturbance-induced changes in vegetation composition and structure are likely to influence mycorrhizal fungi diversity, rodent diversity, and their interactions (House & Bever 2018; Sapsford et al. 2021,Torre et al., 2022). However, the strength and direction of these effects remain unclear, representing a critical knowledge gap with significant forest management implications. In this study, we aimed to address this knowledge gap by 1) assessing the relationship between land use change and mycorrhizal fungal diversity (i.e., richness, composition, and presence of indicator species) in soil and rodent scat samples; 2) comparing the diversity of mycorrhizal fungi in the scats of different rodent genera; 3) analysing the effect of seasonality (dry and wet seasons) on the mycorrhizal fungal diversity present in rodent scats. We predicted that land use change will cause a reduction in the species diversity of AMF and EMF communities resulting in a decrease of diversity in disturbed sites (sites where land use change has occurred) due to the associated plant communities. Secondly, we predicted that responses to land use change would vary among rodent genera. Thirdly, we predicted that seasonality would affect the diversity of mycorrhizal fungi differentially for AMF and EMF, with a stronger seasonal influence on the diversity of EMF than AMF, due to the shorter seasonal production of EMF fruiting bodies.

## Methods

### Study area

**Fig. 1:**
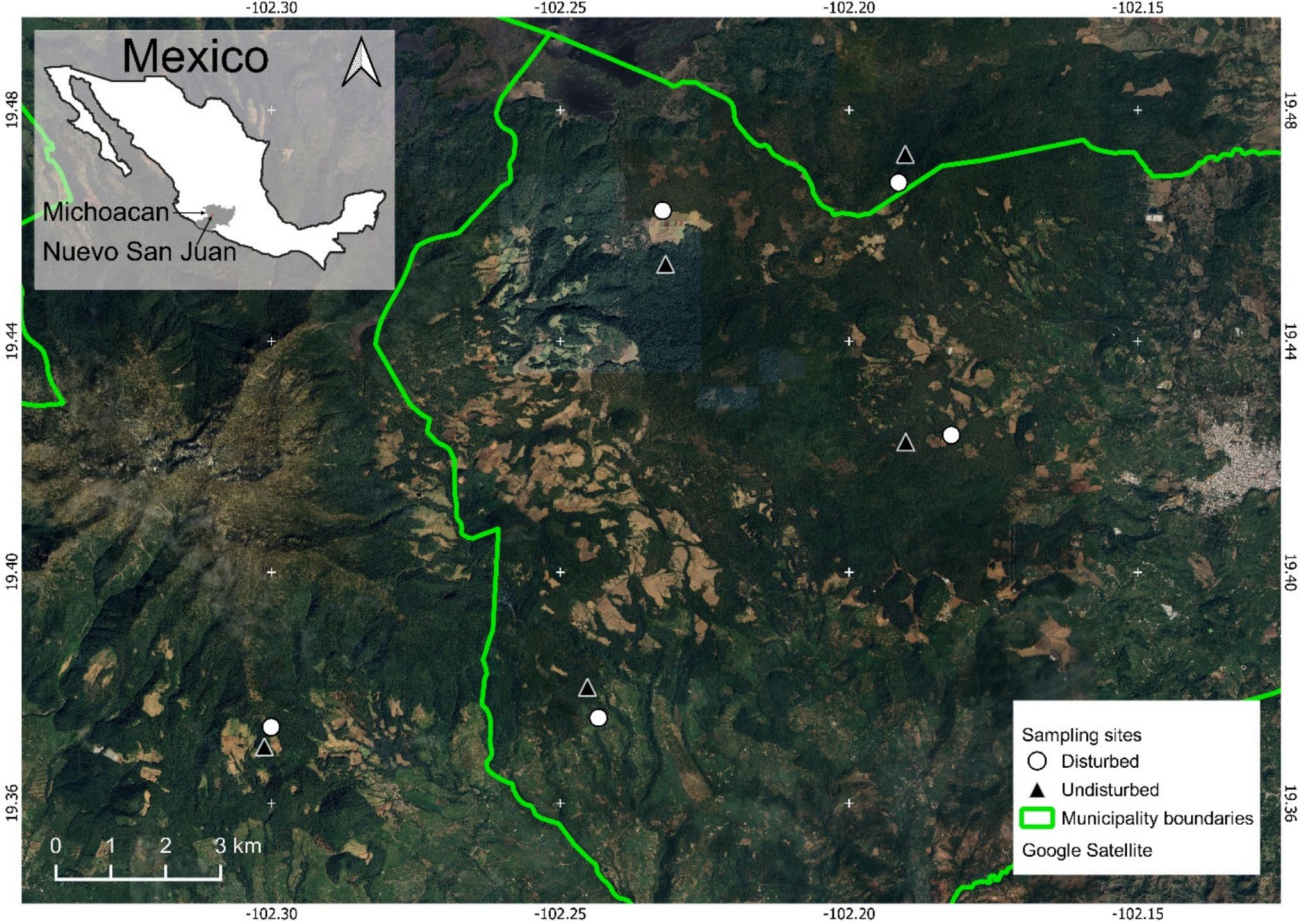
Paired study sites (i.e., disturbed *vs.* undisturbed) sampled to analyse mycorrhizal fungi diversity of soil and rodent scats in Nuevo San Juan, Michoacan, Mexico

This study was conducted in the municipality of Nuevo San Juan Parangaricutiro, Michoacan, Mexico (Fig. 1). This municipality surrounds of a young volcano, El Paricutín which emerged in 1943 and has impacted the soil and vegetation composition and dynamics ever since (Inbar et al. 1994; Medina García et al. 2000). For instance, tree growth was slowed down by up to 42% after the emergence of the volcano due to the deposit of a thick layer of volcanic ash over a radius of at least 10 km (Allende et al. 2022). Soils at our sites are classified as Andosols and Regosols, characterised by low stoniness percentage, good drainage, acidic pH, and a low degree of saturated bases (Allende et al. 2022). The native tree communities in the area are dominated by *Abies, Pinus*, and *Quercus* (Fregoso et al. 2001), all of which are known to form associations with EMF (Molina et al. 1992; Garay–Serrano et al. 2018). Multiple Ericaceae species are also found in the area, such as *Arbutus* spp., *Gaultheria cordata* and *Vaccinium geminiflorum,* which are known to form ericoid mycorrhizal associations (Molina et al. 1992; Medina García et al. 2000). Furthermore, multiple herbaceous species, which are known to form associations with AMF are present in the area; some common taxa include *Artemisia, Ageratina glabrata, Baccharis conferta, Bidens, Cirsium, Gnaphalium*, *Lupinus *spp.,* Rubus ulmifolius, Salvia mexicana*, and *Sonchus* (Bello González and Salgado Garciglia 2007; Bello-González et al. 2015; Shi et al. 2017; Malygin et al. 2021; Argüelles-Moyao et al. 2022; Wu et al. 2023; Vázquez-Santos et al. 2024). The area has experienced extensive land-use changes due to the recent establishment of avocado orchards (Bravo-Espinosa et al. 2014; Latorre-Cárdenas et al. 2023) and a long history of large-scale logging (Velázquez et al., 2015). These widespread land use changes have altered the hydrological cycles of the area (Bravo-Espinosa et al. 2014; Fregoso et al. 2001), exacerbating the impacts of droughts (Dobson et al., 2021).

We selected ten sites in the area to assess the effects of land use change on mycorrhizal fungal communities and their associations with putative rodent dispersers. We followed a paired design, where five of the sites were located under continuous forest cover that have experienced limited changes in land use (undisturbed), each in proximity to a site that had undergone significant land use change (disturbed) (see Gil-Fernández et al. 2024 for further details). The land use change in the disturbed sites occurred before 2004 for four of the sites and in 2017 for one of the sites. The sites within each pair (i.e., one disturbed and one undisturbed site) had a minimum distance of 300 m between them, which is the largest homing distance of *Sigmodon hispidus*, the species with the largest home range among those captured (Cameron and Spencer 1981, 1985). The minimum distance between pairs of sites was 3 km. At each site, forest structure was assessed by measuring tree height, diameter, density, and species diversity in three systematically placed Gentry transects of 50 m × 2 m (Perkins et al. 2019).

### Soil sampling

Soil samples were collected during the rainy season (August 2022). We did not collect samples of soil in the dry season because seasonal changes would be mainly observed in the phenology of sporocarps, and the best way to detect this would be through scats. Furthermore, based on DNA barcoding analyses, fungal communities could appear stable in the soil between seasons due to the presence of relict DNA (Carini et al. 2016). Each sample consisted of ten soil cores taken with a 5 cm diameter sterilised PVC tube buried at a depth of 5 cm, where the highest concentration of mycorrhizal fungi is usually found (Tedersoo et al. 2014). At each site, we collected soil samples associated with smooth-bark Mexican pine (*Pinus pseudostrobus*), multiple species of shrubs, or bare soil. We collected the samples in this manner to standardise the methods of collection throughout the sites, however, the sampled material could be related to multiple species present in the area. For pine and shrub soil samples, we selected five individual plants spaced at least 20 m from each other within the rodent trapping grid (see below). We then collected two soil cores at 30 cm from the base of each individual plant. For the bare soil samples, we collected a pair of soil cores, every 20 m, along the closest access path until completing ten cores. Each collected sample was homogenised in an ethanol-sterilised 2 L container for 2 min. Afterwards, a subsample of each homogenised soil sample was stored in a 1.5 mL Eppendorf tube for DNA extraction.

### Rodent scat sampling

Sampling was conducted in the dry (May 2022) and wet (August 2022) seasons. Paired disturbed and undisturbed sites were simultaneously sampled for two nights. We set up 50 Sherman traps at each sampling site in a 5 × 10 grid arrangement (spaced at 10 m). The traps were baited with peanut butter, oats and vanilla essence. We completed a total sampling effort of 1,997 trap nights. Each captured mammal was photographed and measured in the field for later identification. When present, fresh scats were collected from the bottom of Sherman traps and stored in 1.5 mL Eppendorf tubes. All materials and traps were then sterilised by spraying with 100% ethanol and then flamed to avoid cross-contamination between samples.

### Sample processing and bioinformatics

Scat and soil samples were stored in a portable cooler box in the field and transferred to a − 4 °C refrigerator within 3 h of collection (on average). We extracted DNA from all samples using the DNeasy PowerSoil Pro Kit (Qiagen®) following the manufacturer's protocol. For soil samples we used 250 mg of each subsample. When enough scat material was present, we used 250 mg, however, some samples were had less material. The quality of the extracted DNA was checked using a NanoDrop 2000 spectrophotometer (Thermo Scientific). DNA was dehydrated in a void vacuum to avoid degradation during shipping. The samples were submitted to MR DNA (www.mrdnalab.com, Shallowater, TX, USA) for amplification and sequencing. A segment of the internal transcribed region, ITS1, was amplified using the primer set ITS1F (5'-CTTGGTCATTTAGAGGAAGTAA-3') and ITS2R (5'-GCTGCGTTCTTCATCGATGC-3'). Amplification was done using a 30-cycle PCR under the following conditions: initial denaturation at 95 °C for 5 min, followed by 30 cycles of denaturation at 95 °C for 30 s, annealing at 53 °C for 40 s, and elongation at 72 °C for 1 min, followed by a final elongation step at 72 °C for 10 min. After amplification, PCR products were checked on a 2% agarose gel to confirm amplification success and the relative intensity of PCR products. Samples were multiplexed using unique dual indices and were pooled together in equal amounts based on their molecular weight and DNA concentrations. Pooled samples were purified using calibrated Ampure XP beads, and purified PCR products were used to prepare an Illumina DNA library. Sequencing was performed on an Illumina MiSeq following the manufacturer’s guidelines. The resulting DNA sequences were processed using the MR DNA analysis pipeline (MR DNA, Shallowater, TX, USA). Sequences shorter than 150 bp and sequences with ambiguous base calls were removed. Sequences were quality filtered using a maximum expected error threshold of 1.0 and dereplicated. The unique sequences were denoised. Afterwards, reads with sequencing or PCR point errors were removed, followed by chimera removal. This provided denoised sequences or zero-radius operational taxonomic units (zOTUs). All zOTUs were taxonomically classified using the Basic Local Alignment Search Tool (BLAST) in the National Center for Biotechnology Information (NCBI) Genbank online repository (www.ncbi.nlm.nih.gov). We verified the geographic distribution of each identified mycorrhizal fungi species by consulting the Global Biodiversity Information Facility (www.gbif.org) and scientific literature.

### Functional classification of mycorrhizal fungi

The species or genera identified with > 97% homology were classified into functional guilds using the FUNGuild database (Nguyen et al. 2016) and the *FUNGuildR* R package (Furneaux and Song 2021; R Core Team 2024)*.* We only retained EMF and AMF taxa with a confidence ranking of “probable” or “highly probable”. We also identified rare instances of exclusively ericoid mycorrhizal fungi (i.e., not ectomycorrhizal) in our samples; however, these were not included in the analyses. We used a Hellinger transformation of the DNA sequencing read counts for beta diversity analyses using the *microbiome* R package (Lahti and Shetty 2019; R Core Team 2024).

### Diversity of mycorrhizal fungi in response to land use change

All the below-mentioned analyses were performed separately for our AMF and EMF datasets. Only the samples collected in the wet season (August 2022) were used for these analyses, as we only collected soil samples for this season. We compared the AMF and EMF species richness between site types (disturbed *vs*. undisturbed) and sample types (soil *vs*. scat) with ANOVAs or Kruskal–Wallis tests when assumptions of residual normality and homoscedasticity were not fulfilled. We also compared these groups through rarefaction/extrapolation curves and calculated sample coverage based on the raw incidence of taxa using the *iNext* R package (Hsieh et al. 2016; R Core Team 2024). We compared the diversity between site and sample types using Hutcheson t-tests on Shannon Diversity Indexes (H’), calculated based on the presence counts across samples for AMF and EMF performed in the *ecolTest* R package (Salinas and Ramirez-Delgado 2021; R Core Team 2024).

Differences in the community composition of AMF and EMF between site and sample types were visualised using nonmetric multidimensional scaling (NMDS) ordinations based on the Bray–Curtis dissimilarity index (Hopkins et al. 2021; Oksanen et al. 2022; R Core Team 2024). We checked the homogeneity of multivariate dispersion using the ‘betadisper’ function in the *vegan* R package (Oksanen et al. 2022; R Core Team 2024). To assess differences in the community composition of AMF and EMF between site and sample types, we performed a one-way permutational multivariate ANOVA (PERMANOVA) using the ‘adonis2’ function in *vegan* R package (Oksanen et al. 2022; R Core Team 2024) with 9,999 permutations, setting the random seed to 100. To further explore the composition of the mycorrhizal fungal communities, we identified species of AMF and EMF that are differentially associated, in terms of abundance, with each site type and sample type combinations (i.e., indicator species). We identified the indicator species using the *indicspecies* R package (De Cáceres and Legendre 2009; R Core Team 2024) with 9,999 permutations, setting the random seed 100.

### Diversity of mycorrhizal fungi associated with different rodent genera

We compared the species richness, H’ values, community composition, and indicator species of AMF and EMF species between the three most frequently trapped rodent genera (*Microtus*, *Reithrodontomys*, and *Sigmodon*) using the same analyses as for site type comparisons. For any significant differences, we ran a post hoc analysis using the Dunn test with Bonferroni correction to separate means.

### Diversity of mycorrhizal fungi in rodent scats compared by season

We compared the species richness, H’ values, community composition, and indicator species of AMF and EMF between the dry (May 2022) and the wet (August 2022) seasons using the same analyses as for site type comparisons.

## Results

We identified 8,633 zOTUs, based on 9,118,806 quality-filtered DNA sequences obtained from 129 samples (soils = 28, scats = 101). After classifying the fungi by functional guild, we identified 112 putative mycorrhizal fungi taxa: 21 AMF, 86 EMF, and five ericoid mycorrhizal fungi taxa. Ninety-three (83%) of the mycorrhizal fungi taxa have been previously reported in Mexico (See supplementary materials S1). Seventeen (15%) of the remaining taxa have been recorded in North America. All the soil samples contained AMF and EMF. Seventy-six scat samples contained AMF (75%), while all scat samples contained EMF (100%). When comparing sample types, the AMF species richness was higher in soil samples than in scat samples (χ^2^ = 37.11, d.f. = 1, *p* < 0.001). Similarly, there was a significant difference between the richness of EMF in scat *vs*. soil samples (χ^2^ = 41.83, d.f. = 1, *p* < 0.001). Soil and scat samples had significant differences in dispersion for AMF between them (PERMDISP; F = 31.35, *p* < 0.001). The EMF communities were significantly different when compared by sample type (PERMANOVA; F = 24.96, R^2^ = 0.2, *p* < 0.001).

### Diversity of mycorrhizal fungi in response to land use change

**Fig. 2:**
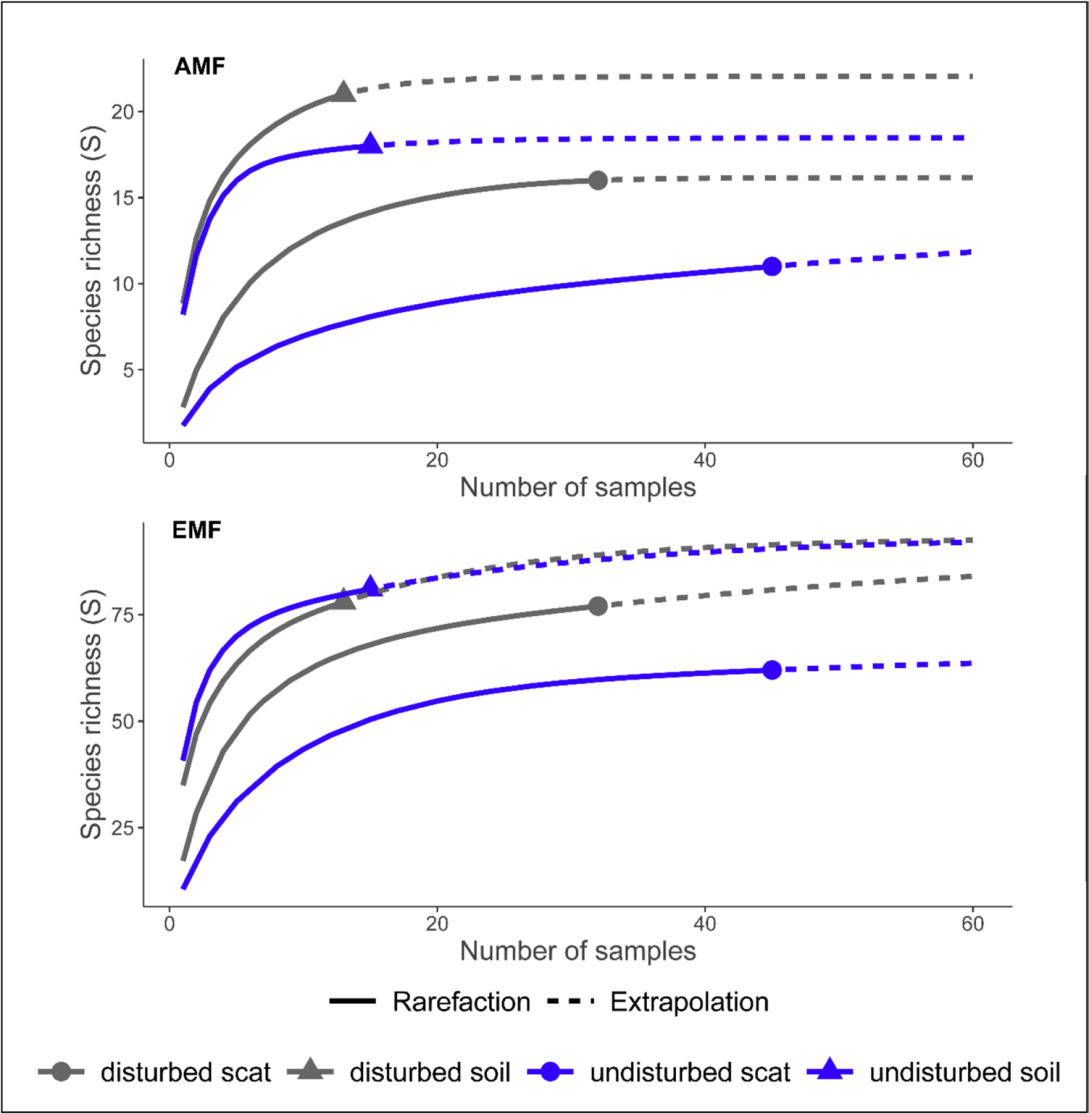
Species richness for arbuscular mycorrhizal fungi (AMF) and ectomycorrhizal fungi (EMF) in rodent scat and soil samples sampled during the wet season in disturbed and undisturbed sites in Nuevo San Juan, Michoacan, Mexico

We processed the data from 15 soil samples from undisturbed sites and 13 from disturbed sites, collected in the wet season. Of these, eight were collected close to *Pinus pseudostrobus* individuals, 10 were in proximity to different shrub species, and 10 were from bare soil. For the comparison between site types, we only included the 77 rodent scats collected in the wet season, 45 samples from undisturbed sites, and 32 from disturbed sites. Of these, 57 contained AMF (35 from undisturbed sites and 22 from disturbed sites). Soil samples from disturbed sites had the highest cumulative AMF richness, whereas the cumulative EMF richness was higher in undisturbed soil samples than in disturbed soil samples (Fig. 2). Arbuscular mycorrhizal fungal richness of soil samples from disturbed and undisturbed sites was not significantly different (F = 0.2, *p* = 0.66). In contrast, scats collected in disturbed sites had a higher AMF richness than those from undisturbed sites (χ^2^ = 5.64, d.f. = 1, *p* = 0.02, Fig. 2). Soil samples collected in undisturbed sites had a higher EMF richness than those collected in disturbed sites (F = 7.17, *p* = 0.01; Fig. 2). In contrast, there were no significant differences in the EMF richness of scat samples collected in the disturbed and undisturbed sites (χ^2^ = 0.3, d.f. = 1, *p* = 0.58). There was no significant difference in the H’ of AMF of soil samples from disturbed and undisturbed sites (F = 0.02, *p* = 0.9), nor for EMF (F = 1.74, *p* = 0.2). In rodent scat samples, the H’ of AMF was significantly higher in disturbed sites (χ^2^ = 5.7, d.f. = 1, *p* = 0.02), but not for EMF (χ^2^ = 0.18, d.f. = 1, *p* = 0.67). When compared by sample type, the H’ of AMF (χ^2^ = 33.28, d.f. = 1, *p*-value < 0.001) and EMF (χ^2^ = 44.06, d.f. = 1, *p* < 0.001) were significantly different.

**Fig. 3:**
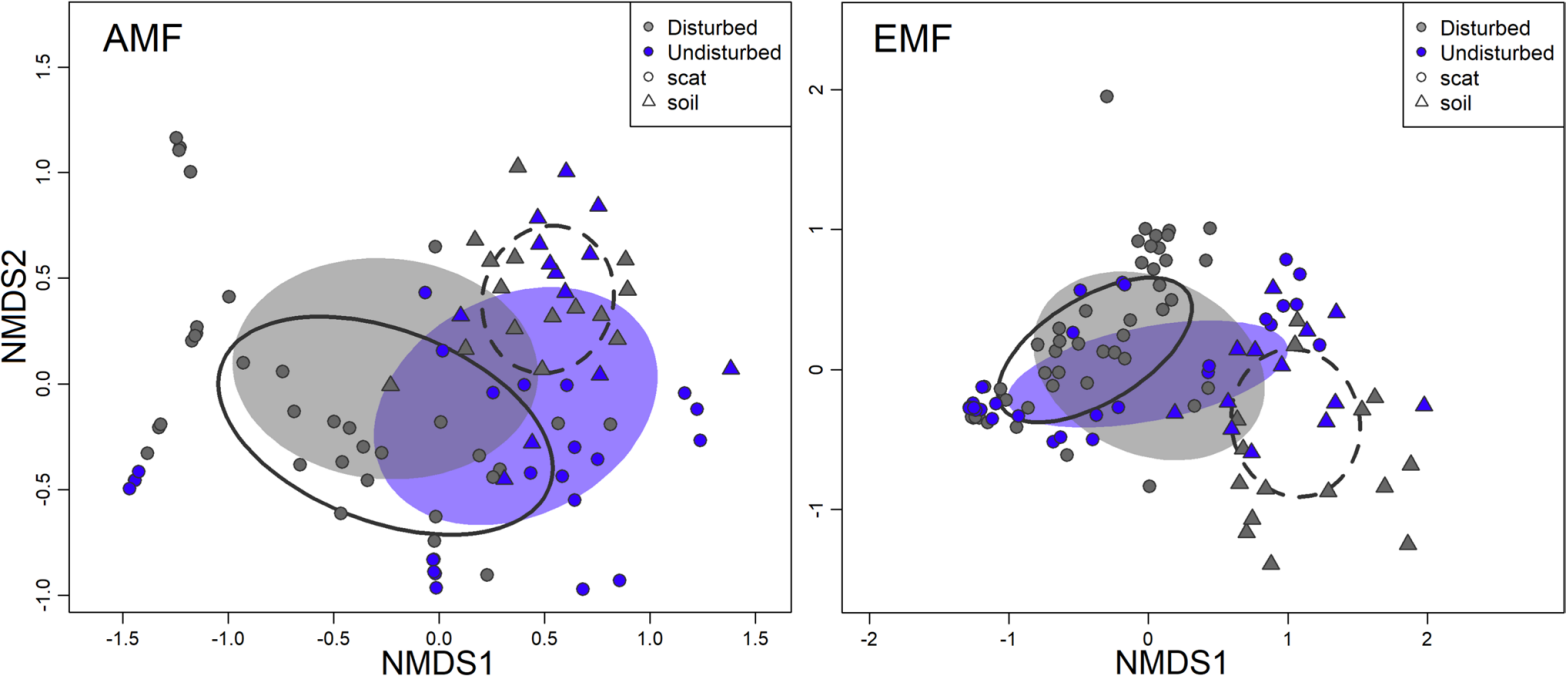
Non-metric multidimensional scaling plots of arbuscular mycorrhizal (AMF) and ectomycorrhizal (EMF) fungal communities found in rodent scat and soil samples compared between disturbed and undisturbed sites of Nuevo San Juan, Michoacan, Mexico. The dashed line ellipses represent soil samples, and the solid line ellipses represent scat samples

The NMDS comparing AMF species in soil and scat samples by site type yielded a fair fit (stress = 0.16, R^2^ = 0.88, Fig. 3). No differences were found in the composition of the AMF communities between disturbed and undisturbed sites in soil samples (PERMANOVA; F = 1.1, R^2^ = 0.04, *p* = 0.33). However, the AMF community composition of rodent scat samples significantly differed between disturbed and undisturbed sites (PERMANOVA; F = 5.68, R^2^ = 0.09, *p* < 0.001). The NMDS comparing the EMF species in soil and scat samples by site type yielded a fair fit (stress = 0.12, R^2^ = 0.94, Fig. 3). In the case of EMF, there was a significant difference between disturbed and undisturbed soil samples (PERMANOVA; F = 2.46, R^2^ = 0.09, *p* = 0.01). Scat samples from disturbed and undisturbed sites showed no significant differences in the composition of EMF communities (PERMANOVA; F = 4.96, R^2^ = 0.06, *p* = 1). The most prevalent AMF taxa varied by sample type. For soils, *Rhizophagus clarus* was the most prevalent taxon in both site types and was present in all the samples. The most prevalent AMF species for scats was *Diversispora versiformis* in both disturbed and undisturbed sites and was present in 47% and 44% of the samples, respectively. Six EMF species were present in all soil samples across both site types: *Inocybe nitiduscula, Rhizopogon salebrosus, Russula cf. roseipes, Suillus cf. variegatus, Tarzetta catinus,* and *Tomentella badia*. Additionally, all soil samples from undisturbed sites harboured *Elaphomyces muricatus* and *Russula queletii*. In scat samples, *Rhizopogon salebrosus* was the only EMF species present in all samples across site types (see supplementary material S1).

When considering the Hellinger transformed DNA sequencing read numbers of the samples, soil samples from disturbed sites had six AMF indicator species, whereas undisturbed soil samples had two indicator species (Table 1, see Supplementary Materials S2 for a complete list). As a group, soil samples (both from disturbed and undisturbed sites) had nine AMF indicator species. Scats from undisturbed sites had one AMF indicator species, while scats collected in disturbed sites did not have any indicator species. Scats and soil samples collected in undisturbed sites shared one AMF indicator species. Regarding EMF, soil samples from disturbed sites had 13 indicator species, whereas soil samples from undisturbed sites had 40 indicator species (see supplementary material S2). Soil samples as a group had nine EMF indicator species. There was one EMF indicator species in scats collected from disturbed sites, and no indicator species were found in scats from undisturbed sites. Scats as a group (both from disturbed and undisturbed sites) had one EMF indicator species *Rhizopogon salebrosus* (Table 1).

**Table 1:** Examples of indicator species per group of variables (site type and sample type) of arbuscular (AMF) and ectomycorrhizal fungi (EMF) from rodent scats and soil samples collected in Nuevo San Juan, Michoacan, Mexico

Sample group	AMF^#^	EMF^#^
	Indicator species	Indicator species
Disturbed/soil	*Acaulospora laevis*	*Cortinarius lacteus*
	*Diversispora aurantia*	*Gymnomyces cf. subfulvus*
	*Entrophospora infrequens*	*Lactarius deliciosus*
	*Redeckera fulva*	*Russula cf. archaea*
	*Cetraspora gilmorei*	*Tarzetta cupularis*
Undisturbed/soil	*Acaulospora paulinae*	*Amanita flavipes*
	*Cetraspora pellucida*	*Gyroporus castaneus*
		*Inocybe nitidiuscula*
		*Piloderma fallax*
		*Russula cerolens*
Disturbed/scats	*-*	*Genea hispidula*
Undisturbed/scats	*Scutellospora nodosa*	-
Soil samples	*Ambispora leptoticha*	*Clavulina cinerea*
	*Diversispora versiformis*	*Inocybe godeyi*
	*Funneliformis mosseae*	*Tomentella stuposa*
	*Glomus cf. dimorphicum*	*Tuber separans*
	*Rhizophagus clarus*	*Wilcoxina rehmii*
Scat samples	*-*	*Rhizopogon salebrosus*
Undisturbed/scats + undisturbed/soil	*Diversispora cf. eburnea*	

^#^A maximum of five indicator species per group are shown. A complete list of AMF and EMF indicator species is provided as Supplementary Material S2

### Diversity of mycorrhizal fungi associated with different rodent genera

For this section, we included the 101 scat samples collected across the dry and wet seasons. The most captured genera of rodents were *Microtus*, *Reithrodontomys*, and *Sigmodon* (Table 2), and the following statistical analyses were done for these three genera only*.* Despite its lower sample size, *Sigmodon* scats had the highest total AMF richness. *Reithrodontomys* scats had the highest EMF richness. However, the mean species richness by sample was also highest for *Sigmodon*, both for AMF and EMF. There was a significant difference in AMF richness between rodent genera (χ^2^ = 15.32, d.f. = 2, *p* < 0.001). These significant differences were between *Sigmodon* and *Microtus* (Z = −3.11, *p* = 0.003), as well as *Sigmodon* and *Reithrodontomys* (Z = −3.88, *p* < 0.001). Overall, *Sigmodon* had the highest richness compared to the other genera (Table 2). No significant differences in the AMF richness were found between *Microtus* and *Reithrodontomys* (Z = 0.29, *p* = 1). The EMF richness significantly differed between rodent genera (χ^2^ = 20.17, d.f. = 2, *p* < 0.001). Within these, *Sigmodon* had a higher EMF richness than *Reithrodontomys* (Z = −4.2, *p* < 0.001, Table 2), and *Microtus* had a higher EMF richness per sample than *Reithrodontomys* (Z = 2.32, *p* = 0.03, Table 2). No significant differences in EMF richness were found between the scats of *Sigmodon* and *Microtus* (Z = −2.06, *p* = 0.06).

**Table 2:** Mycorrhizal fungi species richness and Shannon’s Diversity Index (H’) found in scats of the rodent genera captured in Nuevo San Juan, Michoacan, Mexico. The mean values are calculated based on the individual samples. The total richness represents the cumulative richness

Rodent genus	n	AMF total richness	Mean AMF richness ± SD	Mean AMF H’ ± SD	EMF total richness	Mean EMF richness ± SD	Mean EMF H’ ± SD
*Hodomys*	4	3	1.3 ± 0.6	0.22 ± 0.39	15	5.8 ± 3	0.57 ± 0.36
*Microtus*	18	9	2.8 ± 1.9	0.74 ± 0.68	61	18.6 ± 15*	1.5 ± 0.97
*Peromyscus*	7	4	2 ± 1.4	0.54 ± 0.77	32	7.7 ± 9.3	0.76 ± 0.98
*Rattus*	1	1	1	0	9	9	1.14
*Reithrodontomys*	61	17	2.6 ± 2.1	0.69 ± 0.63	76	10.6 ± 10.2	1.06 ± 1
*Sigmodon*	10	18	5.6 ± 1.4*	1.58 ± 0.28*	60	31.9 ± 9.6*	2.47 ± 1.04*

*AMF* Arbuscular mycorrhizal fungi

*EMF* Ectomycorrhizal fungi

*SD* Standard deviation

*Significantly different compared to the other genera.

The H’ values of the AMF significantly differed between rodent genera (χ^2^ = 14.13, d.f. = 2, *p* < 0.001), with *Sigmodon* scats harbouring higher AMF H’ than *Microtus* (Z = −3.05, *p* = 0.003) and *Reithrodontomys* (Z = −3.7, *p* < 0.001, Table 2). No significant differences were found in H’ values for AMF between the scats of *Reithrodontomys* and *Microtus* (Z = 0.16, *p* = 1). In the case of EMF, there was also a significant difference between rodent genera (χ^2^ = 14.84, d.f. = 2, *p* < 0.001), with scats of *Sigmodon* having a higher EMF H’ than scats of *Reithrodontomys* (Z = −3.62, *p* < 0.001, Table 2). No significant differences were found in EMF H’ between the scats of *Reithrodontomys* and *Microtus* (Z = 1.94, *p* = 0.08), nor between *Sigmodon* and *Microtus* (Z = −1.81, *p* = 0.11).

**Fig. 4:**
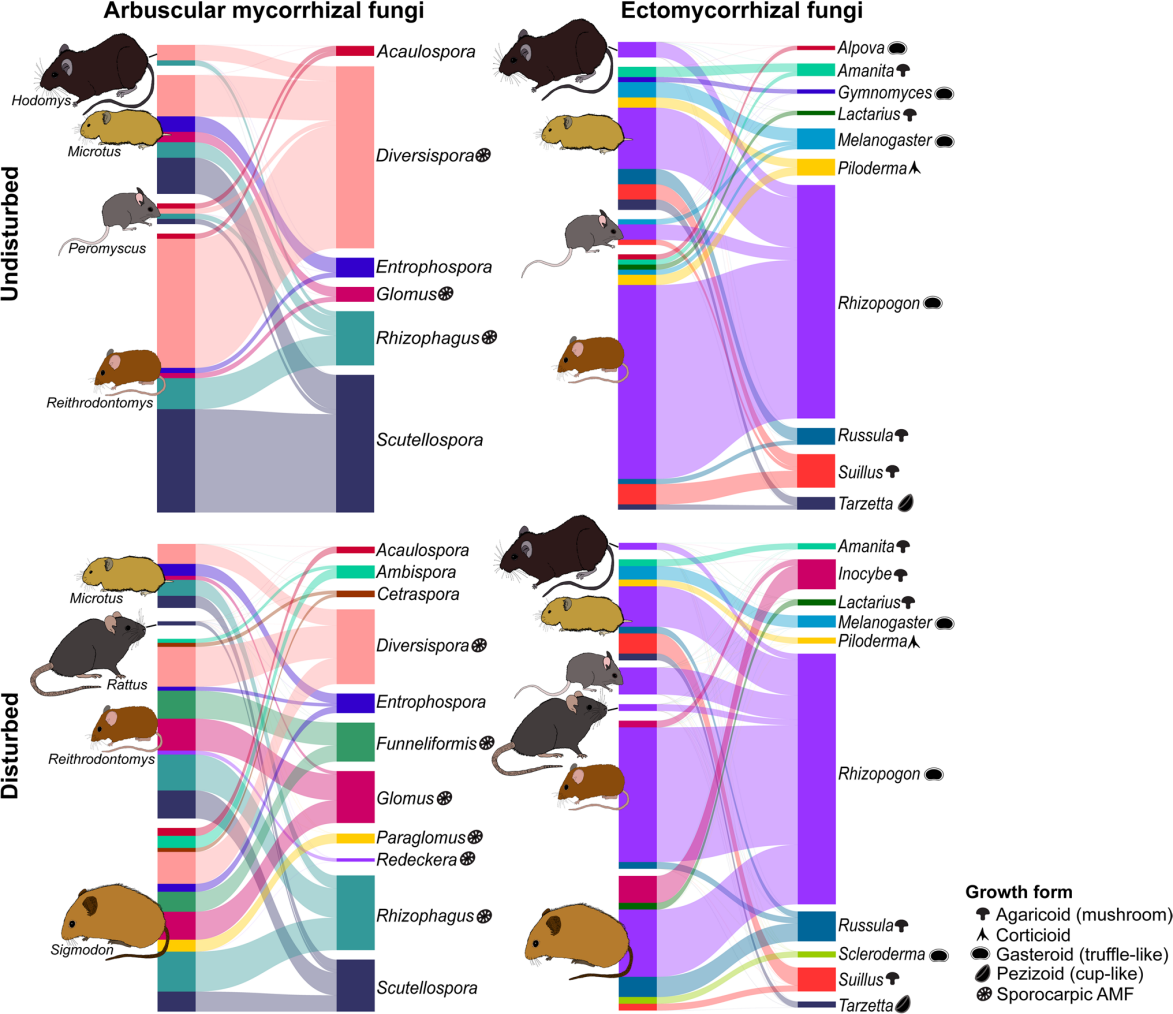
Bipartite graphs of mycorrhizal fungal genera (each right) found in the scats of different rodent genera (each left) captured in disturbed and undisturbed sites of Nuevo San Juan, Michoacan, Mexico

When comparing the mycorrhizal fungal community composition between rodent genera there were no significant differences between the AMF communities (PERMANOVA; F = 2.57, R^2^ = 0.07, *p* = 1). There was a significant difference in the dispersion of the EMF communities between rodent genera (F = 3.91, PERMDISP; *p* = 0.02). Five indicator AMF species were identified for *Sigmodon* and one for *Microtus* (supplementary materials S2). One indicator AMF species was identified for both *Sigmodon* and *Microtus*. No indicator AMF species were identified for *Reithrodontomys.* Twelve EMF indicator species were identified for *Microtus*, 33 for *Sigmodon*, and five for *Sigmodon* and *Microtus* combined. No indicator EMF species were identified for *Reithrodontomys* (supplementary materials S2)*.* When comparing by site types, *Reithrodontomys, Peromyscus* and *Microtus* were associated with a higher number of AMF genera in disturbed sites than in undisturbed sites (Fig. 4). The associations between each rodent species and AMF, as well as EMF species, can be found in Supplementary material S1.

### Diversity of mycorrhizal fungi in rodent scats compared by season

**Fig. 5:**
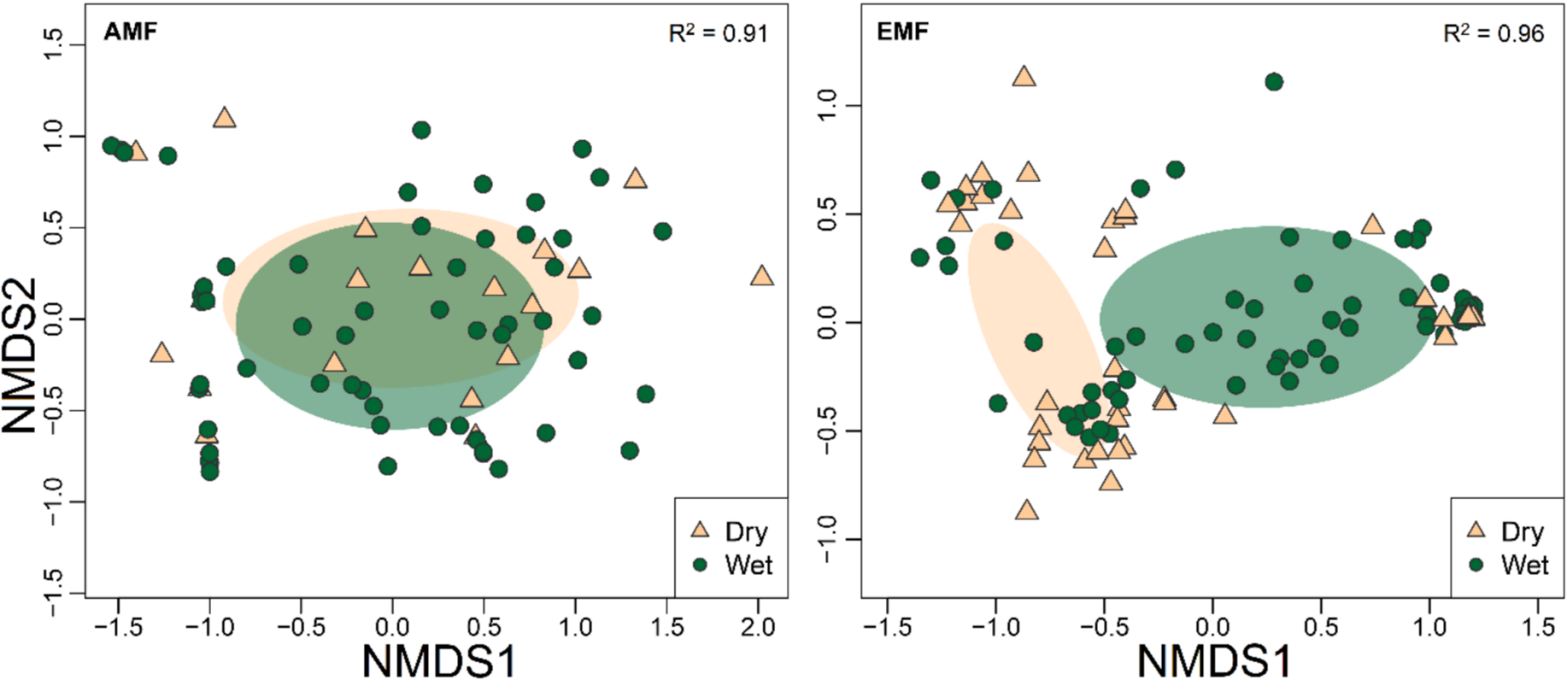
Non-metric multidimensional scaling plots of mycorrhizal fungal community composition found in rodent scats collected in Nuevo San Juan, Michoacan, Mexico for arbuscular mycorrhizal fungi (left; AMF) and ectomycorrhizal fungi (right; EMF) grouped by season

Scat samples collected in the wet season had a cumulative richness of 18 AMF species (sample coverage = 0.99) and 80 EMF species (sample coverage = 0.99). Scat samples collected in the dry season had a cumulative richness of 15 AMF species (sample coverage = 0.88) and 60 EMF species (sample coverage = 0.97). Scat samples collected in the wet and dry seasons did not differ in AMF richness (χ^2^ = 0.002, d.f. = 1, *p* = 0.96) or EMF richness (χ^2^ = 0.04, d.f. = 1, *p* = 0.83). There were also no significant differences in the H’ of AMF in rodent scats between seasons (χ^2^ = 0.8, d.f. = 1, *p* = 0.78). However, the H’ of EMF in scats collected in the wet season was significantly higher than that of scats collected in the dry season (χ^2^ = 5.8, d.f. = 1, *p* = 0.02). The NMDS comparing AMF communities in rodent scats between seasons had a fair fit (stress = 0.14, R^2^ = 0.91). We found no differences in the composition of AMF when comparing dry and wet seasons in both site types (PERMANOVA; F = 0.66, R^2^ = 0.009, *p* = 0.67). The NMDS comparing EMF communities in rodent scats between seasons also had a fair fit (stress = 0.1, R^2^ = 0.96, Fig. 5). For EMF, there was a significant difference in fungal community dispersion between wet and dry seasons (PERMDISP; F = 5.33, *p* = 0.02). One indicator AMF species was identified in the dry season (*Funneliformis mosseae*), but none were identified for the wet season. Five EMF indicator species were identified for the dry season (*Amanita pachycolea, Clavulina rugosa, Inocybe calamistrata, Lactarius deliciosus,* and *Russula queletii*) and two for the wet season (*Inocybe mixtilis* and *Rhizopogon salebrosus*). Thirteen species of AMF were shared between the wet and dry season. The wet season had five unique AMF species, and the dry season had two unique species. Fifty-eight EMF species were shared between seasons. However, there were 22 EMF species found only in the wet season and two species in the dry season.

## Discussion

Rodents play a crucial role as dispersers of mycorrhizal fungi (Maser et al. 1978; Paz et al. 2021); however, this interaction may be influenced by factors such as land use change, rodent identity, and seasonality. We aimed to assess how the interactions between rodents and mycorrhizal fungi are affected by these factors. We found that forest mycorrhizal fungal communities associated with soil and rodents were affected by land use change. As we predicted, the direction of change in diversity and composition of these communities depended on the functional ecology of the mycorrhizal fungi (i.e., AMF or EMF) and the sample type (soil or rodent scats). Soil samples from disturbed and undisturbed sites significantly differed in EMF richness and composition but showed no difference in AMF richness. Contrastingly, scat samples from disturbed and undisturbed sites differed in AMF richness, diversity (H’), and composition. Soil samples consistently showed higher AMF and EMF richness and H’ than scat samples. We found significant differences in EMF and AMF richness and H’ between *Microtus*, *Sigmodon* and *Reithrodontomys,* supporting our prediction that mycorrhizal fungi diversity in scat samples would vary between different rodent dispersal vectors. Ectomycorrhizal fungi diversity (H’) varied seasonally, while AMF diversity (H’) did not, which partly supports our prediction that the effect of seasonality on the diversity would be greater on EMF than AMF. Ongoing microscopy work confirms the presence of EMF (e.g., *Rhizopogon, Russula, Lactarius* and *Suillus*) and AMF (e.g., *Glomus*) spores in our rodent scat samples (González-Medina, et al., unpublished data), supporting their potential role as dispersal vectors of these fungi at our study sites.

Although some studies have shown declines in soil AMF diversity due to disturbance (House and Bever 2018; Amalia et al. 2021), others have found that these responses are highly context-dependent, where an increase in diversity or no change has also been observed in response to disturbance (Jonsson et al. 1999; González-Cortés et al. 2012; Pereira et al. 2014; van der Heyde et al. 2017; Xu et al. 2017; Carrillo-Saucedo et al. 2018; Sepp et al. 2018). The lack of observed differences in AMF communities in soil samples from disturbed and undisturbed sites could be explained by the large area coverage and severity of the disturbance in our study area (Latorre-Cárdenas et al. 2023). In general, herbaceous plants such as successional shrub and herb species, primarily form AMF associations (Lembrechts et al. 2014; van der Heijden et al. 2015; Stephens et al. 2021; Okada and Matsuda 2022; Clavel et al. 2024). In our undisturbed sites, the influence of the disturbance matrix, composed of avocado orchards and logged forest areas, could facilitate the presence of generalist herbaceous plants, such *as Ageratina glabrata*, *Lupinus* spp., *Roldana angulifolia*, and *Salvia mexicana*; as well as the invasive species *Rubus ulmifolius,* which are known to form associations with AMF (Shi et al. 2017; Correia et al. 2019; Malygin et al. 2021; Argüelles-Moyao et al. 2022; Wu et al. 2023; Vázquez-Santos et al. 2024). Moreover, the presence of relict AMF DNA or AMF spore banks could also explain the similar diversity of AMF we found in soil samples from different sites, as this type of DNA could be sequenced and obscure the results (Carini et al. 2016; Silva-Flores et al. 2019). In contrast, scat samples from disturbed sites exhibited higher AMF diversity, likely reflecting a higher consumption of plant roots of successional shrubs and herbaceous species or the ingestion of AMF sporangia associated with these plants in disturbed than in undisturbed sites by rodents (Andersen 1987; Matamoros Trejo and Cervantes 1992; Heth et al. 2002; Meikle and Powers 2011). Shrubs and herbaceous plants were more abundant in our disturbed sites than in undisturbed sites (see Gil-Fernández et al. 2024). This would result in more AMF DNA making its way through the rodent's digestive tract and ending up in scat, but not soil, samples from disturbed sites.

We found low numbers of AMF indicator species per site type and sample type, which accounts for a low representative AMF diversity (Dufrene and Legendre 1997), as well as for the primers we used for PCR amplification and DNA sequencing (Stockinger et al. 2010). In soil samples, one of the AMF indicator species in both disturbed and undisturbed sites was *Rhizophagus clarus,* which is explained by the fact that this is a common species with a cosmopolitan distribution (Stürmer et al. 2025). The symbiotic benefits of this species have been studied in agricultural ecosystems, particularly concerning drought tolerance (Oliveira et al. 2022) and its use in bioremediation (Rafique et al. 2019). Therefore, in disturbed sites, this species may assist vegetation resilience to drought (Messa and Savioli 2021). Although AMF diversity differed between scats from disturbed and undisturbed sites, a single AMF indicator species identified from scats was found only in undisturbed sites. This indicator species was *Scutellospora nodosa*, which has been linked to a significant increase in phosphorus uptake and growth by plants (Rewcastle 2005).

The presence of EMF is essential for some plant species to grow and for their seedling establishment (Ashkannejhad and Horton 2006; Finlay 2008; Hayward et al. 2015), especially in temperate coniferous forests, such as in our study region, that harbour many plant species that form obligate associations with EMF. We found that soils from undisturbed sites had higher EMF richness than soils from disturbed sites, likely due to the absence of native forest plants such as pine trees in the latter (Aguirre et al. 2021; Bowd et al. 2022). Further, disturbance could also directly impact soil properties that influence the prevalence of EMF, such as pH, humidity and porosity (Gryndler et al. 2010; Santolamazza-Carbone et al. 2023). As an example, the EMF fruiting bodies in the disturbed sites would be exposed to harsher environmental conditions, such as increased sun exposure and lower soil moisture (e.g., Luoma et al. 2003). Interestingly, although the soil samples from the undisturbed sites had significantly higher EMF richness than the soil samples from disturbed sites at the sample level, both site types had a similar cumulative EMF richness. This could be explained by the presence of fungal spore banks, owing to the longevity of EMF fungal spores in soils (Okada and Matsuda 2022), spore dispersal by invertebrates, birds, and large vertebrates, besides rodents, as well as secondary dispersal through predation (Ponder 1980; Paz et al. 2021; Elliott et al. 2023). In contrast to the soil samples, we found no difference in EMF diversity in scat samples from disturbed and undisturbed sites. However, every sample did contain EMF, highlighting the potential importance of rodent-mediated dispersal of mycorrhizal fungi in providing native seedlings with access to EMF in disturbed and undisturbed areas (Stephens and Rowe 2020).

In contrast to the low numbers of indicator species identified for AMF across sample and site types, we found 40 EMF indicator species in association with undisturbed soil samples. This high number of EMF indicator species could be explained by the more stable conditions of undisturbed areas (Correia et al. 2021), and the presence of their specialised hosts such as oak trees (*Quercus* spp.) and conifers (Argüelles-Moyao and Garibay-Orijel 2018; Balami et al. 2021). In rodent scats, the only EMF indicator species found was *Rhizopogon salebrosus*, which is an obligate symbiont of pine trees (family Pinaceae), and it was present in every rodent scat sample. *Rhizopogon* species are known to be dispersed mainly in mammal scats (Ashkannejhad and Horton 2006; Peay et al. 2012) and have been widely documented as a dominant taxon in rodent scats (Luoma et al. 2003; Stephens et al. 2021; Bradshaw et al. 2022). Therefore, given the accumulating evidence supporting the role of rodents acting as dispersers of mycorrhizal fungi in disturbed environments, it is reasonable to infer that rodents play an important role in the establishment and regeneration of native pine stands in disturbed sites in our study area (Allen et al. 1992; Cázares and Trappe 1994; Terwilliger and Pastor 1999; Ashkannejhad and Horton 2006; Nuñez et al. 2013; Vlk et al. 2020; Aguirre et al. 2021; Stephens et al. 2021; Policelli et al. 2022).

It has been noted that different rodent genera generally consume similar mycorrhizal fungi (Bradshaw et al. 2022) and that individual rodents can consume different species of fungi in a short period of time (Luoma et al. 2003). We found a high incidence of both AMF (75%) and EMF (100%) in rodent scat samples (also see Nuske et al. 2019). The higher incidence of EMF likely reflects their attractiveness as food sources to rodents (as aromatic mushrooms or truffle-like fruiting bodies). Nevertheless, some of the recorded AMF species (i.e., *Diversispora versiforme, Funneliformis mosseae, Glomus macrocarpum, Paraglomus, Redeckera fulva,* and *Rhizophagus clarus*) found in our rodent scat samples form sporocarps, which are also attractive to mammals (McGee and Baczocha 1994; Oehl et al. 2011; Krings et al. 2015). Although all the rodents we captured consumed mycorrhizal fungi, we found significant differences in the fungal communities in the scats of the most captured genera. *Sigmodon hispidus* was associated with a significantly higher mean species richness and H’ per sample of both AMF and EMF compared with all other rodent genera. It also had the highest number of indicator EMF species (33) compared with other rodents. These indicator species included diverse growth forms such as agaricoid, pezizoid, clavarioid, and boletoid, suggesting that *S. hispidus* is a generalist mycophagous species. The high diversity of mycorrhizal fungi in *Sigmodon* scats can be explained by its ground-dwelling habit, larger size, and larger home range compared to the other rodents we studied. To our knowledge, this is the first record of members of this genus as a consumer of mycorrhizal fungi. *Sigmodon hispidus* is a pest of crops, and it is heavily controlled by farmers (Hermira and Michalski 2022).

The second most important rodent, in terms of AMF and EMF richness and H’, was *Microtus mexicanus*, which has been previously identified as a disperser of mycorrhizal fungi (Trappe and Maser 1976). This species also had the second-highest number of indicator EMF species (12), indicating mycophagy and a preference for certain EMF species. *Microtus mexicanus* and *Sigmodon hispidus* shared some EMF indicator species, which could be explained by the similarity of their habits, being both ground-dwellers (Cameron and Spencer 1981; Núñez 2005; Ceballos and Oliva 2005). The third most important rodent genus was the most-captured *Reithrodontomys*. There are records of *Reithrodontomys* species as dispersing mycorrhizal spores via ingestion (Frank and Southworth 2006; Elliott et al. 2022b). Although less represented in our study, the rodent genera *Peromyscus* and *Rattus* have been widely documented as dispersers of mycorrhizal spores (Castillo-Guevara et al. 2011; Pérez et al. 2012; Elliott et al. 2022b). To our knowledge, we are providing the first evidence for *Hodomys alleni* as a disperser of both AMF and EMF. In agreement with previous studies (e.g., Bradshaw et al. 2022), we found a high overlap in the mycorrhizal fungi taxa consumed by the rodents that we captured.

As expected, the rodent scats collected in the wet season had a higher H’ of mycorrhizal fungi than those collected in the dry season. This seasonal variation in mycorrhizal fungi diversity is well-known (Mangan and Adler 2002; Kataržytė and Kutorga 2011; Nuske et al. 2019) and it is related to the higher abundance of fruiting bodies, and thus food sources for rodents, in the wet season (Kataržytė and Kutorga 2011). The timing of this increase in ingestion of mycorrhizal fungi is important due to the masting period that occurs during the rainy season, where the presence of these fungi would be essential for the establishment of pine seedlings (Stephens and Rowe 2020). However, EMF H’ was the only diversity metric that differed between seasons. The lack of difference in the richness of EMF, as well as the higher number of EMF indicator species for the dry season, could reflect the phenology of the fruiting bodies (Johnson 1994). Also, this could reflect that if there are fruiting bodies in the dry season (in May), these would be consumed as soon as they are available (Elliott, et al. 2022a, b). The lack of difference in AMF diversity across seasons that we observed could be because the AMF abundance at our sites, and therefore their availability for consumption, is affected by other variables, including soil chemical properties such as pH and N content (Han et al. 2023).

To our knowledge, our study provides one of the most extensive analyses of the diversity of mycorrhizal fungi associated with rodents in Mexico. However, ex situ studies have assessed the effects of rodent digestion on the viability of mycorrhizal fungi spores, where different effects on mycorrhization of host plants have been found (D’Alva et al. 2007; Castillo-Guevara et al. 2011; Pérez et al. 2012). We found that the DNA of AMF and EMF was highly prevalent in rodent scats, and, coupled with a high incidence of EMF and AMF spores in our scat samples (González-Medina, et al., unpublished data), this indicates that rodents are important dispersal vectors of mycorrhizal fungi at our study sites. In the context of land use change, rodent-mediated dispersal is one of the mechanisms that might allow the build-up of mycorrhizal fungal spore banks in disturbed areas, in turn benefiting forest regeneration, especially by native plant species that rely on EMF symbionts. Thus, managing rodent-fungal interactions could be a strategy in ecological restoration aimed at rehabilitating degraded forests by facilitating the dispersal of native mycorrhizal fungi. Future research on spore counts, spore viability, and scat inoculation trials will provide valuable information to further understand the role of rodent dispersal of both AMF and EMF. It is also worth noting that, as our study area has been under drastic anthropogenic pressure (e.g., logging and agriculture), rodents that are forest specialists may be absent or rare (we only recorded eight out of the 30 rodent species known from the area; Ceballos & Oliva 2005). This points to the need for increased rodent monitoring in the area to fully understand the dispersal dynamics of mycorrhizal fungi in temperate forests in the Anthropocene.

## Supplementary Information

Below is the link to the electronic supplementary material.

Supplementary file1 (XLSX 61 KB)

Supplementary file2 (DOCX 37.2 KB)

## Data Availability

Data and code that support the findings of this study have been deposited in DRYAD repository URL: https://datadryad.org/stash/share/ESiJ79BUqt88YRH9LW6HFo38e4wqjNsxeyTbUI1yNhU.
