# Supplementary material for: The impact of land use change on mycorrhizal fungi and their associations with rodents: insights from a temperate forest in Mexico
Source: Mycorrhiza. 2025 May 8;35(3):36. doi: 10.1007/s00572-025-01210-x (PMC12062193; doi:10.1007/s00572-025-01210-x)
Supplement: Supplementary file 2 — Supplementary file2 (DOCX 37.2 KB) [file 572_2025_1210_MOESM2_ESM.docx]

**The impact of anthropogenic disturbance on mycorrhizal fungi and their associations with rodents: insights from a temperate forest in Mexico**

Margarita Gil-Fernández^1, 2, 3^*, Alexandra J. R. Carthey^1^, Eduardo Mendoza^4^, Oscar Godínez-Gómez^5^, M. Cristina MacSwiney G.^6^, Arnulfo Blanco-García^7^, Christian A. Delfín-Alfonso^3^, Johannes J. Le Roux^1^

^1^School of Natural Sciences, Macquarie University, New South Wales, 2109, Australia

^2^Posgrado en Biología Integrativa, Instituto de Investigaciones Biológicas, Universidad Veracruzana. Luis Castelazo Ayala Avenue, Industrial Ánimas, Xalapa, Veracruz, 91190, Mexico

^3^Laboratorio de Vertebrados, Instituto de Investigaciones Biológicas, Universidad Veracruzana. Luis Castelazo Ayala Avenue, Industrial Ánimas, Xalapa, Veracruz, 91190, Mexico

^4^Instituto de Investigaciones sobre los Recursos Naturales, Universidad Michoacana de San Nicolás de Hidalgo, San Juanito Itzicuaro Avenue, Nueva Esperanza, Morelia, Michoacán, 58330, México.

^5^Department of Wildlife Ecology and Conservation, University of Florida, Gainesville, FL 32618, United States of America

^6^Centro de Investigaciones Tropicales, Universidad Veracruzana, José María Morelos y Pavon 44, Centro, Xalapa, Veracruz, 91000, México

^7^Facultad de Biología, Universidad Michoacana de San Nicolás de Hidalgo. Francisco J. Múgica Avenue, Ciudad Universitaria, Morelia, Michoacan, 58060, Mexico

*Corresponding author: margarita.gil-fernandez@hdr.mq.edu.au

**Supplementary material 2.** Indicator species of arbuscular fungi (AMF) and ectomycorrhizal fungi (EMF) from rodent scats and soil samples collected in Nuevo San Juan, Michoacan, Mexico.

Table 1. Indicator species per group of variables (site type and sample type) of arbuscular (AMF) and ectomycorrhizal (EMF) fungi from rodent scats and soil samples collected in Nuevo San Juan, Michoacan, Mexico.

| Sample group | AMF |  |  | EMF | |  |  |
| --- | --- | --- | --- | --- | --- | --- | --- |
|  | Indicator species | Stat | p | Indicator species | Stat | | p |
| Disturbed/soil | *Entrophospora infrequens* | 0.54 | 0.0001 | *Russula archaea* | 0.422 | | 0.0001 |
|  | *Acaulospora laevis* | 0.455 | 0.0002 | *Russula roseipes* | 0.34 | | 0.0001 |
|  | *Diversispora aurantia* | 0.451 | 0.0007 | *Tarzetta cupularis* | 0.414 | | 0.0003 |
|  | *Acaulospora spinosa* | 0.346 | 0.0025 | *Gymnomyces subfulvus* | 0.377 | | 0.0015 |
|  | *Scutellospora gilmorei* | 0.294 | 0.0206 | *Cortinarius lacteus* | 0.268 | | 0.005 |
|  | *Redeckera fulvum* | 0.284 | 0.0238 | *Lactarius deliciosus* | 0.278 | | 0.0059 |
|  |  |  |  | *Scleroderma bovista* | 0.318 | | 0.0061 |
|  |  |  |  | *Tuber zhongdianense* | 0.292 | | 0.0185 |
|  |  |  |  | *Imaia gigantea* | 0.313 | | 0.02 |
|  |  |  |  | *Lactarius chrysorrheus* | 0.294 | | 0.0256 |
|  |  |  |  | *Scleroderma areolatum* | 0.253 | | 0.0325 |
|  |  |  |  | *Gilkeya compacta* | 0.257 | | 0.0348 |
|  |  |  |  | *Russula insignis* | 0.264 | | 0.0499 |
| Undisturbed/soil | *Acaulospora paulinae* | 0.457 | 0.0002 | *Amanita flavipes* | 0.416 | | 0.0001 |
|  | *Scutellospora pellucida* | 0.282 | 0.0098 | *Gyroporus castaneus* | 0.554 | | 0.0001 |
|  |  |  |  | *Inocybe nitidiuscula* | 0.489 | | 0.0001 |
|  |  |  |  | *Piloderma fallax* | 0.517 | | 0.0001 |
|  |  |  |  | *Russula cerolens* | 0.449 | | 0.0001 |
|  |  |  |  | *Inocybe mixtilis* | 0.457 | | 0.0001 |
|  |  |  |  | *Amanita fulva* | 0.383 | | 0.0001 |
|  |  |  |  | *Tarzetta catinus* | 0.642 | | 0.0001 |
|  |  |  |  | *Tomentella badia* | 0.614 | | 0.0001 |
|  |  |  |  | *Tomentella ferruginea* | 0.463 | | 0.0001 |
|  |  |  |  | *Amanita multisquamosa* | 0.529 | | 0.0002 |
|  |  |  |  | *Amanita muscaria* | 0.381 | | 0.0002 |
|  |  |  |  | *Elaphomyces muricatus* | 0.42 | | 0.0002 |
|  |  |  |  | *Phellodon confluens* | 0.438 | | 0.0002 |
|  |  |  |  | *Inocybe auricoma* | 0.328 | | 0.0003 |
|  |  |  |  | *Phylloporus pelletieri* | 0.48 | | 0.0003 |
|  |  |  |  | *Russula brevipes* | 0.312 | | 0.0003 |
|  |  |  |  | *Boletus rubellus* | 0.387 | | 0.0004 |
|  |  |  |  | *Piloderma olivaceum* | 0.349 | | 0.0011 |
|  |  |  |  | *Inocybe ochroalba* | 0.387 | | 0.0012 |
|  |  |  |  | *Russula queletii* | 0.352 | | 0.0016 |
|  |  |  |  | *Tomentellopsis zygodesmoides* | 0.349 | | 0.002 |
|  |  |  |  | *Amanita xylinivolva* | 0.298 | | 0.0041 |
|  |  |  |  | *Russula variata* | 0.276 | | 0.0058 |
|  |  |  |  | *Suillus brevipes* | 0.283 | | 0.0058 |
|  |  |  |  | *Russula lutea* | 0.326 | | 0.0067 |
|  |  |  |  | *Pachyphloeus carneus* | 0.339 | | 0.0094 |
|  |  |  |  | *Russula pectinatoides* | 0.346 | | 0.0117 |
|  |  |  |  | *Tomentella subclavigera* | 0.313 | | 0.012 |
|  |  |  |  | *Clavulina rugosa* | 0.323 | | 0.016 |
|  |  |  |  | *Boletus aestivalis* | 0.308 | | 0.0185 |
|  |  |  |  | *Hebeloma collariatum* | 0.29 | | 0.0196 |
|  |  |  |  | *Russula nigricans* | 0.274 | | 0.0325 |
|  |  |  |  | *Russula emetica* | 0.271 | | 0.0328 |
|  |  |  |  | *Suillus volcanalis* | 0.288 | | 0.0371 |
|  |  |  |  | *Inocybe adaequata* | 0.284 | | 0.0373 |
|  |  |  |  | *Amanita pachycolea* | 0.251 | | 0.0377 |
|  |  |  |  | *Cortinarius olivaceofuscus* | 0.256 | | 0.0415 |
|  |  |  |  | *Morchella crassipes* | 0.263 | | 0.0432 |
|  |  |  |  | *Thelephora americana* | 0.275 | | 0.0436 |
| Disturbed/scats | *-* | *-* | *-* | *Genea hispidula* | 0.284 | | 0.0295 |
| Undisturbed/scats | *Scutellospora nodosa* | 0.379 | 0.0076 | *-* | - | | - |
| Soil samples | *Rhizophagus clarus* | 0.603 | 0.0001 | *Tomentella stuposa* | 0.535 | | 0.0001 |
|  | *Glomus dimorphicum* | 0.541 | 0.0001 | *Clavulina cinerea* | 0.419 | | 0.0007 |
|  | *Diversispora versiformis* | 0.514 | 0.0002 | *Inocybe godeyi* | 0.39 | | 0.0008 |
|  | *Glomus macrocarpum* | 0.46 | 0.0007 | *Tuber separans* | 0.464 | | 0.0008 |
|  | *Glomus indicum* | 0.37 | 0.0075 | *Tomentella bryophila* | 0.386 | | 0.0008 |
|  | *Ambispora leptoticha* | 0.344 | 0.01 | *Wilcoxina rehmii* | 0.4 | | 0.001 |
|  | *Funneliformis mosseae* | 0.322 | 0.0287 | *Suillus variegatus* | 0.336 | | 0.0088 |
|  | *Rhizophagus intraradices* | 0.308 | 0.035 | *Inocybe lanatodisca* | 0.338 | | 0.0113 |
|  | *Rhizophagus irregularis* | 0.304 | 0.0427 | *Tricholoma myomyces* | 0.31 | | 0.0192 |
| Scat samples | *-* | *-* | *-* | *Rhizopogon salebrosus* | 0.575 | | <0.0001 |
| Undisturbed/scats + undisturbed/soil | *Diversispora eburnea* | 0.316 | 0.0236 |  |  | |  |

Table 2. Indicator species per group of variables rodent vector genus of arbuscular (AMF) and ectomycorrhizal fungi (EMF) from rodent scats collected in Nuevo San Juan, Michoacan, Mexico.

| Rodent vector genus | AMF | | EMF | |
| --- | --- | --- | --- | --- |
|  | Indicator species | p | Indicator species | p |
| *Microtus* | *Entrophospora infrequens* | 0.0428 | *Russula lutea* | 0.0001 |
|  |  |  | *Russula cerolens* | 0.0004 |
|  |  |  | *Amanita muscaria* | 0.0005 |
|  |  |  | *Russula variata* | 0.0009 |
|  |  |  | *Russula nigricans* | 0.0017 |
|  |  |  | *Amanita fulva* | 0.0027 |
|  |  |  | *Boletus rubellus* | 0.0032 |
|  |  |  | *Piloderma olivaceum* | 0.0048 |
|  |  |  | *Amanita flavipes* | 0.0065 |
|  |  |  | *Inocybe auricoma* | 0.0091 |
|  |  |  | *Suillus brevipes* | 0.0097 |
|  |  |  | *Cortinarius glaucopus* | 0.0218 |
| *Sigmodon* | *Glomus macrocarpum* | 0.0018 | *Russula xerampelina* | 0.0001 |
|  | *Paraglomus brasilianum* | 0.0021 | *Clavulina rugosa* | 0.0001 |
|  | *Funneliformis mosseae* | 0.0041 | *Inocybe calamistrata* | 0.0001 |
|  | *Ambispora leptoticha* | 0.0066 | *Wilcoxina rehmii* | 0.0001 |
|  | *Rhizophagus intraradices* | 0.0019 | *Russula roseipes* | 0.0001 |
|  |  |  | *Inocybe lanatodisca* | 0.0001 |
|  |  |  | *Russula queletii* | 0.0001 |
|  |  |  | *Tarzetta cupularis* | 0.0001 |
|  |  |  | *Russula brevipes* | 0.0002 |
|  |  |  | *Amanita pachycolea* | 0.0002 |
|  |  |  | *Tomentella ferruginea* | 0.0002 |
|  |  |  | *Pachyphloeus carneus* | 0.0002 |
|  |  |  | *Porphyrellus pseudoscaber* | 0.0003 |
|  |  |  | *Scleroderma areolatum* | 0.0003 |
|  |  |  | *Inocybe nitidiuscula* | 0.0005 |
|  |  |  | *Scleroderma verrucosum* | 0.0005 |
|  |  |  | *Chroogomphus vinicolor* | 0.0007 |
|  |  |  | *Lactarius deliciosus* | 0.0007 |
|  |  |  | *Amanita multisquamosa* | 0.0012 |
|  |  |  | *Hebeloma collariatum* | 0.0018 |
|  |  |  | *Russula archaea* | 0.0018 |
|  |  |  | *Lactarius chrysorrheus* | 0.0025 |
|  |  |  | *Cortinarius lacteus* | 0.0041 |
|  |  |  | *Amanita xylinivolva* | 0.006 |
|  |  |  | *Tricholoma myomyces* | 0.0077 |
|  |  |  | *Genea hispidula* | 0.0158 |
|  |  |  | *Scleroderma polyrhizum* | 0.0168 |
|  |  |  | *Tuber zhongdianense* | 0.0168 |
|  |  |  | *Inocybe rimosoides* | 0.0176 |
|  |  |  | *Inocybe subnudipes* | 0.0209 |
|  |  |  | *Russula compacta* | 0.0209 |
|  |  |  | *Russula pectinatoides* | 0.0253 |
|  |  |  | *Inocybe chelanensis* | 0.0277 |
| *Sigmodon + Microtus* | *Rhizophagus clarus* | 0.0268 | *Tuber separans* | 0.0048 |
|  |  |  | *Piloderma fallax* | 0.0126 |
|  |  |  | *Clavulina cinerea* | 0.0136 |
|  |  |  | *Tomentella badia* | 0.0139 |
|  |  |  | *Elaphomyces muricatus* | 0.0141 |
